# Oxidation-Specific Epitopes in Non-Alcoholic Fatty Liver Disease

**DOI:** 10.3389/fendo.2020.607011

**Published:** 2020-12-09

**Authors:** Tim Hendrikx, Christoph J. Binder

**Affiliations:** ^1^ Department of Molecular Genetics, School of Nutrition and Translational Research in Metabolism (NUTRIM), Maastricht University, Maastricht, Netherlands; ^2^ Department of Laboratory Medicine, Medical University Vienna, Vienna, Austria; ^3^ Research Center for Molecular Medicine of the Austrian Academy of Sciences (CeMM), Vienna, Austria

**Keywords:** oxidative stress, innate immunity, non-alcoholic fatty liver disease, lipid peroxidation, steatohepatitis (NASH), oxidation-specific epitopes

## Abstract

An improper balance between the production and elimination of intracellular reactive oxygen species causes increased oxidative stress. Consequently, DNA, RNA, proteins, and lipids are irreversibly damaged, leading to molecular modifications that disrupt normal function. In particular, the peroxidation of lipids in membranes or lipoproteins alters lipid function and promotes formation of neo-epitopes, such as oxidation-specific epitopes (OSEs), which are found to be present on (lipo)proteins, dying cells, and extracellular vesicles. Accumulation of OSEs and recognition of OSEs by designated pattern recognition receptors on immune cells or soluble effectors can contribute to the development of chronic inflammatory diseases. In line, recent studies highlight the involvement of modified lipids and OSEs in different stages of the spectrum of non-alcoholic fatty liver disease (NAFLD), including inflammatory non-alcoholic steatohepatitis (NASH), fibrosis, and hepatocellular carcinoma. Targeting lipid peroxidation products shows high potential in the search for novel, better therapeutic strategies for NASH.

## Introduction

In parallel with the global epidemic obesity, the prevalence and incidence of non-alcoholic fatty liver disease (NAFLD) has greatly increased over time and continues in doing so, making it the most common liver disease worldwide (1). Nowadays, the prevalence of NAFLD is estimated around 25% in Western countries. NAFLD covers a histological spectrum of liver conditions with varying degrees of liver injury and scarring in individuals who do not consume alcohol in harmful quantities and was first recognized as distinct entity about 40 years ago. NAFLD ranges from excessive hepatic lipid accumulation alone (steatosis) that might progress into non-alcoholic steatohepatitis (NASH), which is characterized by inflammatory cell infiltrates. NASH increases the risk for further liver fibrosis, cirrhosis and hepatocellular carcinoma, ultimately requiring liver transplantation (2). In fact, progressive NASH is the second ranked cause for liver transplantation, directly following alcoholic liver disease (3). Although the diagnosis for NAFLD or NASH can be suspected based on ultrasound imaging and abnormal levels of liver enzymes in serum, NASH can only be definitely diagnosed by liver biopsy and evaluation of a pathologist. While lifestyle interventions and associated weight loss can improve steatosis alone, sustained effects are difficult to obtain and the presence of inflammation makes prognosis considerably worse. Further, although multiple drugs and combination therapies are under investigation, currently no truly effective treatment exists (4, 5). The progression from steatosis alone into NASH can be explained by the so-called “two-hit hypothesis,” in which lipid accumulation would represent the first hit, rendering the tissue more prone for a second hit (such as oxidative stress and lipid peroxidation) triggering inflammation and liver damage. However, this concept changed into a more “multiple parallel hits” theory, in which different molecular triggers simultaneously cause steatosis and liver injury (6–8). More recently, metabolic associated fatty liver disease (MAFLD) has been suggested as a novel term reflecting current knowledge about disease pathology more precisely (9). In this review, we use NASH as term for describing more advanced fatty liver disease compared to steatosis alone in NAFLD.

The occurrence of oxidative stress reactions and the accumulation of reactive oxygen species are common features observed in different stages of the NAFLD spectrum (10, 11). On one hand, excess exposure and build-up of free fatty acids in hepatocytes cause lipotoxicity and damage to mitochondria, and stimulates the release of pro-inflammatory cytokines and microvesicles (12). Further, the activation of non-parenchymal cells, especially resident Kupffer cells, together with the recruitment of infiltrating monocytes and neutrophils to the liver contributes to inflammation *via* the release of cytokines, chemokines, nitric oxide, and reactive oxygen species (2). Importantly, although reactive oxygen species are produced normally as by-products of cellular metabolism, they can attack different vital macromolecules such as proteins, lipids, and nucleic acids (DNA/RNA). This results in different oxidative damage products such as protein carbonyls, lipid peroxides, and 8-hydroxy-2′-deoxyguanosine (8-OH-dG), respectively. These products are often used as biomarkers of oxidative stress and have all been associated with NAFLD/NASH (reviewed in (13)). In order to overcome inappropriate levels of reactive oxygen species, we depend on the scavenging capacity by antioxidants in our system. Relevantly, in parallel to increased oxidative stress, reduced systemic levels of antioxidants such as glutathione or vitamin E, as well as lower anti-oxidative enzyme activity has been documented during NAFLD (13–15). In this review, we will particularly focus on the oxidation of membrane lipids, a process called lipid peroxidation, and their involvement in steatohepatitis (16).

Besides affecting their biological function, peroxidation of lipids also results in the generation of several degradation end-products which can further modulate the normal properties of proteins and lipids. Furthermore, highly reactive aldehydes generated in the process of lipid peroxidation modify self-molecules and form antigenic adducts, known as oxidation specific epitopes (OSEs), which are bound by various receptors of the immune system to alert the host and promote their removal to prevent inflammatory effects (17, 18). However, accumulation of OSEs and their insufficient clearance triggers sterile inflammation. Major carriers of OSEs are cells undergoing cell death (apoptosis), extracellular vesicles, and damaged lipoproteins, such as oxidized low-density lipoproteins (OxLDL). Owing to their biological activities, OSEs and their immune recognition are involved in a wide variety of physiological and pathological processes, including atherosclerosis (19–21) and several autoimmune diseases such as systemic lupus erythematosus (22). Here, we provide an overview of studies linking OSEs and their immune recognition to NASH and potential implications in the field of fatty liver disease.

### OSE Generation

Various mechanisms cause lipid peroxidation, particularly polyunsaturated fatty acids (PUFAs), which involves both enzymatic and non-enzymatic mechanisms (23). The enzymatic processes of lipid peroxidation include the activation of lipoxygenases, myeloperoxidases, cyclo-oxygenases, and cytochrome p450. On the other hand, non-enzymatic oxidation is done by free radicals, which are indirectly generated by nicotinamide adenine dinucleotide phosphate (NADPH) oxidases and nitric-oxide synthases. Both enzymatic and non-enzymatic mechanisms result in the generation of lipid-hydroperoxide molecules (LOOH), which then decompose. As part of this degradation process a large variety of secondary products including malondialdehyde (MDA), 4-hydroxynonenal (4-HNE), and the remaining core aldehyde of oxidized phospholipids (OxPL) are produced (24, 25). These remaining end-products of lipid peroxidation can further propagate oxidative damage as a result of defective clearance and as such, have been considered as downstream mediators of oxidative stress. Notably, measuring lipid peroxidation degradation products, especially MDA and 4−HNE, by the frequently used 2−thiobarbituric acid reaction (TBAR) assay, is an established method for assessing oxidative stress (16).

PUFA-containing phospholipids are particularly prone to oxidative damage (16, 23). Hence, the major membrane phospholipid phosphatidylcholine is very susceptible to free-radical-induced oxidation, resulting in the exposure of hydrophilic phosphocholine (PC) headgroups and the generation of a complex mixture of OxPL and their terminal degradation products (26). Especially, oxidation of the PUFA chain at the sn−2 position of phosphatidylcholine results in its degradation and the formation of reactive PUFA fragmentation products such as MDA. Further, the oxidation of cholesterol and cholesteryl esters can take place, resulting in structural changes and altered biological activities (27, 28). Owing to their biological activities and pro-inflammatory potential, the formation of lipid peroxidation-derived adducts can be viewed as post-translational modifications generating neo-epitopes which are now recognized as a type of danger-associated molecular pattern (DAMP) (20). Importantly, several of these lipid-derived adducts have been documented and implicated in a wide variety of pathologies, including NAFLD and NASH (18, 29, 30).

### OSE Presence in NAFLD

**Figure 1:**
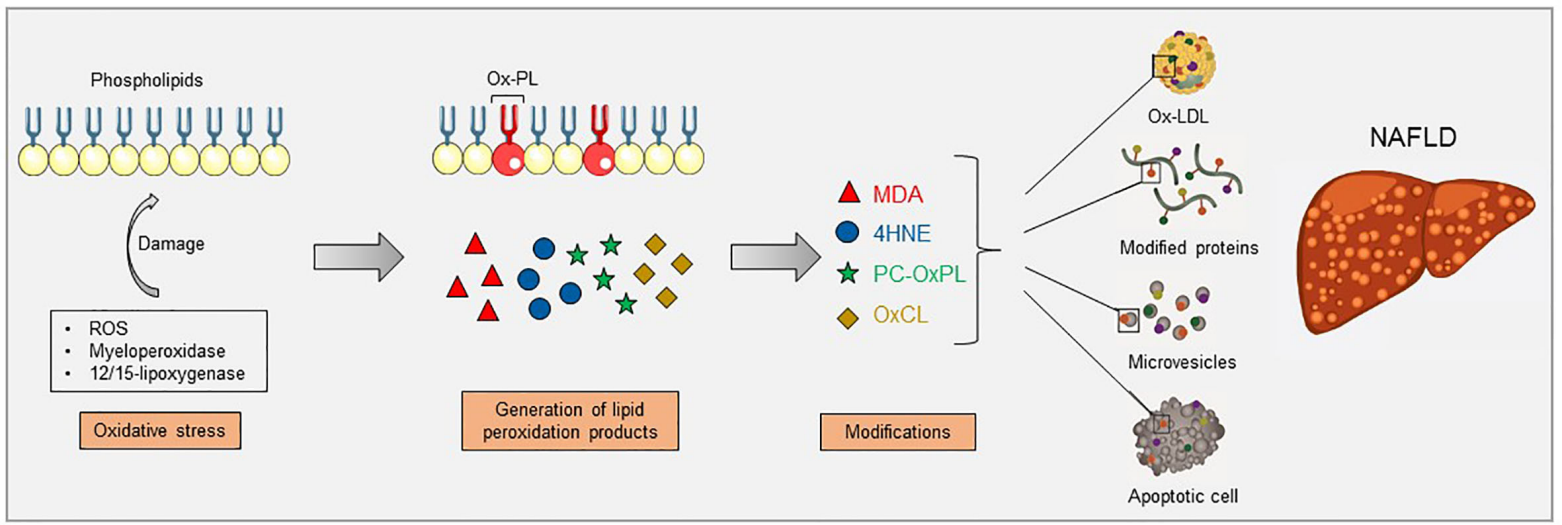
Increased oxidative stress causes lipid peroxidation, which can occur *via* enzymatic reactions, such as myeloperoxidase and 12/15-lipoxygenase, and non-enzymatic reactions, such as reactive oxygen species (ROS). Lipid peroxidation of membrane phospholipids results in their fragmentation and the generation of breakdown products which can further modify free amino groups of proteins and lipids, forming covalent adducts and creating oxidation-specific epitopes (OSEs), including malondialdehyde (MDA), 4-hydroxynonenal (4-HNE), phosphocholine on oxidized phospholipids (PC-OxPL), and oxidized cardiolipin (OxCL). These epitopes are carried by oxidized low-density lipoproteins (OxLDL), modified proteins, microvesicles, and apoptotic cells, aspects that have been shown to be present during NAFLD. This figure was produced using Servier Medical Art (Servier, www.servier.com, licensed under a Creative Commons Attribution 3.0 Unported Licence).

Oxidation of LDL is thought to contribute to fatty liver disease progression by multiple mechanisms, including the formation of OSEs (31, 32). As mentioned before, OSEs are present on apoptotic cells, OxLDL, and microvesicles, components that all have been shown to be associated with NAFLD. Here, we provide several lines of evidence that indicate the presence and importance of different lipid peroxidation products in the onset of NASH (see **Figure 1**).

Among the different aldehydes that can be formed as secondary products during lipid peroxidation, MDA and 4-HNE are the most extensively studied and both are associated with different stages of fatty liver disease. A significant correlation between hepatic 4-HNE adducts and the stage of fibrosis has been described (33), and increased mitochondrial 4-HNE–protein adducts during NASH development have been reported (34). Multiple studies have indicated increased amounts of MDA, as measured by the TBAR assay, and OxLDL levels in human NAFLD and NASH patients in comparison to control subjects (15, 35–39). In addition, the presence of MDA adducts in livers has been documented for several different experimental rat and mouse models of NAFLD and NASH (40–43). In line with these publications, work from our group indicated the presence of MDA adducts in livers of human NASH (44), as well as during steatohepatitis in hypercholesterolemic *Ldlr^−/−^* mice on high-fat high-cholesterol diet (45), a murine model resembling human lipid profiles of NAFLD (46). Using *in vitro* and *in vivo* approaches, we showed that MDA epitopes detectable in hepatic inflammation act as sterile mediators of inflammation *via* their stimulation of cytokine secretion by macrophages and promotion of leukocyte recruitment (45).

Recently, Sun et al. reported elevated amounts of PC-OxPLs in the liver and circulation during NASH compared to controls using various murine models and human subjects (47). In humans, increased plasma and liver PC-OxPL was more closely associated with NASH rather than steatosis, suggesting their importance during disease progression. Mechanistically, OxPLs induced mitochondrial damage and ROS accumulation, partly *via* modification of the anti-oxidative enzyme manganese superoxide dismutase (MnSOD/SOD2), thereby blocking its activity.

Work from our group demonstrated that various OSEs are carried by circulating extracellular vesicles (48), a heterogeneous population of small membrane-deliminated structures including exosomes and microvesicles. Importantly, the release of extracellular vesicles has been identified as a result of hepatocyte lipotoxicity and has been implicated in NAFLD (49). Indeed, toxic lipid classes are abundantly present in circulation of NASH patients and stressed/damaged hepatocytes as a result of treatment with lipotoxic lipids causes the release of large amounts of extracellular vesicles (50). Besides, microvesicles have been proposed as potential biomarkers for chronic liver diseases (51). Microvesicles, potentially hepatocyte-derived, have been shown to stimulate pro-inflammatory responses *via* the activation of immune cells and stellate cells in the liver and thus might link lipotoxicity and lipid peroxidation to inflammation and fibrosis, major components of NASH. More specific microvesicle profiling, including assessment of biological properties and cell-cell interaction, will enable us to better understand the role of OSE-carrying microvesicles during NASH development and progression.

Another phospholipid component prone to oxidation is cardiolipin, which is almost exclusively present at the inner mitochondrial membrane. Cardiolipin is importantly involved in mitochondrial energetics and mitochondrial dependent steps of apoptosis. Hence, oxidized cardiolipin (OxCL) has been associated with mitochondrial dysfunction. Relevantly, increased cardiolipin levels are reported in experimental NASH models as well as human NAFLD (52–54). Moreover, patients with NAFLD have increased circulating IgG antibody titers towards OxCL compared to healthy controls (55), further supporting the presence of OxCL during NAFLD.

### Innate Immune Recognition of OSEs

**Figure 2:**
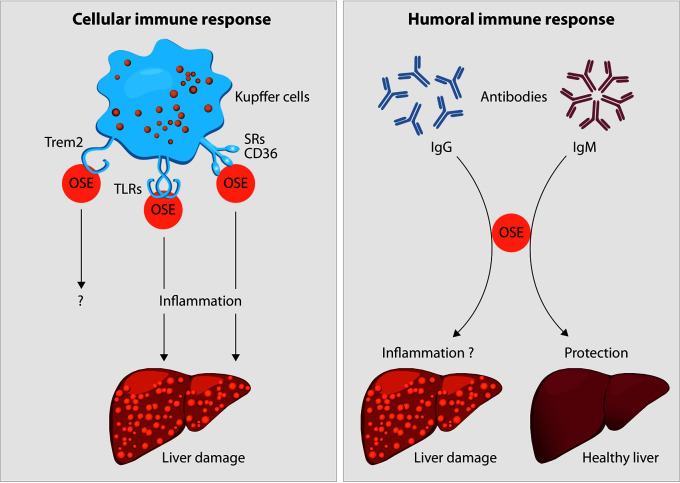
Oxidation-specific epitopes (OSEs) act as danger-associated molecular patterns (DAMPs) which are recognized by different pattern recognition receptors (PRR) as part of the cellular immune response towards OSEs. Receptors known to bind to certain lipid peroxidation adducts are the family of scavenger receptors (SR) such as CD36, Toll-like receptors (TLRs) and the triggering receptor expressed on myeloid cells 2 (TREM2). In the liver, the presence of SR and TLRs on Kupffer cells and their uptake of modified lipids has been shown to cause inflammation, thereby leading to liver damage during NAFLD progression. The functional role of TREM2 during NAFLD has not been addressed yet. Moreover, B cell-derived antibodies are able to recognize OSEs. Whereas increased anti-OSE IgG titers are associated with NASH and fibrosis, potentially *via* triggering pro-inflammatory responses, IgM antibodies targeting OSE are found to be protective against NASH, both by neutralizing the pro-inflammatory effects of OSE and by enhancing the clearance of dying cells.

As described above, lipid peroxidation and its end-products are associated with both structural and functional alterations of these macromolecules. In order to protect us from potential detrimental effects of accumulated altered self-molecules, the human body requires recognition mechanisms to provide effective clearance. Indeed, OSEs are believed to represent a class of DAMPs which can stimulate both adaptive and innate immune responses, dependent on recognition by pattern recognition receptors (PRR) (21, 56). Detailed characterization of various OSEs and the innate immune responses towards them indicated that OSEs are recognized by both cellular and soluble PRRs, which we will discuss in this section in view of steatohepatitis (see **Figure 2**).

#### Cellular Receptors

As part of the innate immune response triggered by OSEs, PRR present on the surface of immune cells, such as the scavenger receptor family, recognize and internalize oxidized but not native LDL particles (57). As such, macrophages mediate the phagocytosis of oxidatively altered molecules, triggering inflammatory cascades *via* the secretion of pro-inflammatory chemokines and cytokines such as CC-chemokine ligand 1 (CCL1), CCL2, and CCL5, thereby promoting increased recruitment of monocytes which further contribute to the production of pro-inflammatory mediators as well as oxidative damage (58). Among the family of scavenger receptors, CD36, SR-A1, and SR-A2 are the best described and most relevant receptors for OxLDL uptake as macrophages lacking CD36, SR-A1, and SR-A2 have 75–90% reduced binding and degradation of OxLDL (59). In addition, we demonstrated that bone marrow-derived macrophages from mice lacking CD36 or SR-A1 secreted less CXCL1 compared to wildtype cells after stimulation with malondialdehyde-acetaldehyde (MAA) epitopes, the immunodominant subset of MDA epitopes (45). Similar to lipid-laden foam cells as occurring during atherosclerotic lesions, Kupffer cells, resident macrophages of the liver, show a foamy appearance after the enhanced uptake of modified lipoproteins during NAFLD (60). Importantly, we found that hematopoietic SR-A1 and/or CD36 deficiency protects against hepatic steatosis and inflammation in hyperlipidemic *Ldlr^−/−^* mice fed an atherogenic diet, indicating that SR-mediated uptake of modified lipids is at least in part responsible for diet-induced inflammation in the liver (61). Further studies indicated that OxLDL uptake by macrophages is associated with abnormal intracellular trafficking, resulting in lysosomal trapping of OxLDL, cholesterol crystal formation, and dysfunctional lysosomes (31). Similar to cholesterol crystals, stimulating J774 macrophages with MDA-modified hen egg lysozyme (MDA-HEL) caused lysosomal rupture (62). Therefore, one might hypothesize that OSE-induced lysosomal damage might account for inflammatory responses and apoptosis during diet-induced liver disease. Indeed, human NASH patients have increased circulatory levels of cathepsins, lysosomal enzymes responsible for breakdown of internalized products, indicating lysosomal leakage (63). Taken together, ample evidence supports the involvement of scavenger receptor-mediated uptake of modified lipids in NAFLD.

Toll-like receptors (TLRs) are another class of PRR taking part in the response to damaged lipoproteins, capable of binding to a variety of different pathogen-associated molecular patterns (PAMPs), including bacterial and viral components, as well as DAMPs. Additionally, certain TLRs have been shown to recognize—as part of a multimeric complexes with other PRRs—OxPL, OxLDL, and other OSEs, thereby mediating pro-inflammatory signals (18). Particularly TLR2, TLR4, and TLR6 have been shown to respond to OSEs, i.e. oxidized cholesteryl esters (OxCE) and OxPL on the surface of extracellular vesicles appear to be ligands for TLR4 (29, 64). In relation to NAFLD, the involvement of certain TLRs in disease progression has been described, such as TLR2, TLR4, TLR7, and TLR9 (65). For instance, absence of TLR4 in *Ldlr^−/−^* mice on atherogenic diet has been shown to protect against triglyceride accumulation in the liver (66) and features of NASH were ameliorated in TLR4 deficient mice receiving methionine choline-deficient (MCD) diet (67). Further, activation of TLR7 signaling was found to reduce lipid accumulation and autophagy in hepatocytes, thereby preventing NAFLD progression. Interestingly, 4-HNE and MDA potentially inhibit this process, thereby aggravating disease onset (68). Nevertheless, a direct link between OSE-TLR signaling and fatty liver disease development is missing as most studies focus on the recognition of bacterial ligands.

More recently, the triggering receptor expressed on myeloid cells 2 (TREM2), belonging to the immunoglobulin superfamily and mainly expressed on myeloid cells, has been shown to bind and recognize lipoproteins and apolipoproteins, including ApoE, LDL, and MDA-LDL particles (69), suggesting TREM2 might also recognize OSEs. Upon cleavage of TREM2 by A Disintegrin And Metalloprotease (ADAM) 10 and ADAM17, soluble TREM (sTREM2) is released (70). Whether sTREM has similar binding capacities as TREM2 is currently not known. Interestingly, single-cell RNA sequencing (scRNAseq) studies have identified the presence of *Trem2* expressing macrophages in several lipid-mediated diseases, including obesity (71) and atherosclerosis (72). Importantly, also in relation to liver damage during fatty liver disease, the emergence of so-called NASH-associated macrophages (NAMs) (73) and scar-associated macrophages (SAMs) (74), that are characterized by high *Trem2* expression, has been described. *Xiong et al.* found more hepatic *Trem2* expression in human and murine NASH and described that increased *Trem2* expression in livers of NASH patients is positively correlated with AST and ALT levels (73). These findings are further supported by another study, which indicated more *Trem2* expressing macrophages in the liver of human subjects with cirrhosis based on scRNAseq analysis of CD45+ cells (74). As the receptor has been reported to bind MDA-LDL, these data suggest a potential novel mechanism of OSE-induced immune recognition. Nevertheless, functional studies assessing the role of TREM2 during the onset of NAFLD are currently missing and more specific insights in recognition of different OSEs by TREM2 and its soluble form are needed.

#### Soluble Receptors

Besides recognition by receptors present on the cell surface, OSEs represent targets for several soluble PRRs that also include secreted forms of cellular PRR. One soluble protein capable of binding OSEs is C-reactive protein (CRP), an acute-phase reactant made in the liver. Originally, CRP was identified as the plasma component binding PC located on the capsular polysaccharide of *Streptococcus pneumoniae*. Only later, CRP was also identified to bind PC-OxPL on OxLDL and dying cells, indicating CRP to respond to a common OSE of microbial origin or derived from lipid peroxidation (75). Although some discrepancies exist over the relationship between CRP levels in NAFLD, most studies report increased circulatory CRP levels, which is a known marker for systemic inflammation, during NAFLD (76). One could hypothesize that changes in CRP levels are correlated with oxidative stress and/or lipid peroxidation products, thereby affecting study outcomes. However, whether CRP itself has a functional role in NAFLD development is unclear.

Further, MDA epitopes have been found to be targets of proteins involved in the complement machinery, which exerts a key function in the maintenance of tissue homeostasis *via* immune surveillance and managing clearance of metabolic waste and dead cells (77). Besides the C3 cleavage product C3a (78), a pro-inflammatory anaphylatoxin, we identified that complement factor H (CFH) recognizes and binds MDA epitopes. CFH is a regulator of complement activity as it inhibits the alternative pathway by preventing C3 cleavage. Whereas increased activation of the complement system is reported in NAFLD (79), the specific levels of CFH mRNA were found downregulated in patients with NASH in line with protein levels of CFH in the liver (80). Therefore, low CFH expression might contribute to the harmful effects of lipid peroxidation products in fatty liver disease. In addition, our group has shown that genetic variants of one of the MDA-binding sites of CFH influence the capacity of CFH to bind MDA (81). Further, we recently demonstrated that genetic deletions of complement factor H-related protein 1 (CFHR1), which was previously shown to bind to MDA-LDL (82), and 3 (CFHR3) affects the ability of plasma CFH to bind MDA surfaces. Moreover, purified CFHR1 and CFHR3 competes with CFH for binding to MDA-epitopes (83). Our findings indicate the influence of genetic variations within the CFH/CFHR1/CFHR3 locus on the recognition and binding of OSEs, thereby affecting outcome in diseases associated with oxidative stress and aging such as NAFLD. Further molecular and genetic studies are needed to elucidate the involvement of CFH and CFHR proteins in relation to OSE recognition during fatty liver disease.

Natural antibodies are pre-existing germline-encoded antibodies that are already present at birth and of which the occurrence does not depend on external antigens, as they can be found in germfree mice. Natural antibodies are predominantly of the IgM type, with a broad specificity to various pathogens, but which are also able to recognize endogenous antigens, such as OxPL and adducts formed by end products of the lipid peroxidation process (84). Besides their protective function in the first line defense against invading microbes, natural IgM antibodies maintain homeostasis *via* the clearance of dying cells and metabolic waste products. Indeed, MDA-specific natural IgM recognize apoptotic cells as well as microvesicles carrying MDA epitopes (48). Natural IgM antibodies are produced by B1 cells, which are widely studied in the field of atherosclerosis and believed to exert protective effects *via* the production of natural IgM antibodies (85). In line with these findings in cardiovascular disease, we found that patients with NAFLD have lower levels of IgM targeting OSEs than healthy individuals (44). In addition, we demonstrated that IgM plasma titers towards an immunological mimotope of MDA, P1 mimotope, inversely correlate with signs of obesity, systemic inflammation, and liver injury. Further studies by others and us indicating the protective role of OSE-IgM in the course of NAFLD are described later on in the section about OSE as targets for treatment options.

Taken together, OSE represent DAMPs that initiate certain immune responses upon recognition by several cellular and soluble PRRs as part of the innate immune system. Besides that, adaptive immune reactions can also be triggered by OSEs.

### Adaptive Immune Recognition of OSEs

In contrast to innate immunity, adaptive immunity is acquired throughout life as a direct consequence of exposure to external antigens. This way, an immense repertoire of highly specific receptors is generated, resulting in lifelong immune memory, which is mediated by B cells and the antibodies they secrete as well as various T cell subsets. The role of adaptive immunity in the progression of NAFLD, including anti-OSE responses, has recently been reviewed elsewhere [see (30)]. Hence, we will only briefly describe OSE-specific B-cell mediated adaptive responses during NAFLD.

In addition to the involvement of anti-OSE IgM in NAFLD, IgG antibodies targeting OSE have been shown to be elevated in ~40% of adults diagnosed with NAFLD or NASH (55) and in 60% of children with NASH (86). Particularly, IgG antibodies against the cyclic MAA adduct are increased in adults with NAFLD or NASH (55). Further, anti-OSE IgG levels are correlated with the degree of lobular inflammation and are an independent predictor of fibrosis. In contrast to natural IgM, anti-OSE IgG are thought to be produced by B2 cells, suggesting the involvement of specific acquired immunity by different B cell subsets in NASH (87). In line, increased IgG titers were found to be associated with increased maturation of liver B2 cells to plasma cells in diet-induced NAFLD in rats as well as in MCD-induced NASH in mice (88). As such, increased B2 cells might be involved in NASH by promoting pro-inflammatory signals and antigen presentation to CD4 T cells and as a source of pathogenic IgG antibodies. The potential opposing effects of B1 and B2 cells (decreased IgM and elevated IgG titers) are similar to observations during atherosclerosis (89). Hence, further studies investigating the role of B1 and B2 cell subsets and their implications in NASH are needed to potentially identify novel therapy options in which specific B cell subsets are targeted.

### OSE as Targets for the Treatment of NAFLD

As oxidative stress is one of the best characterized triggers for liver inflammation during NASH, makes it an attractive target for intervention to prevent disease progression. Although work by us and others point to the potential of targeting lipid peroxidation products and immune recognition of OSEs to ameliorate the inflammatory response occurring in NASH, these studies only involve the usage of different murine models. So far, evidence from human studies in which OSEs or immunity towards OSEs are directly targeted for the treatment of NAFLD and NASH is lacking. Nevertheless, multiple studies exploited the effect of nutrients and antioxidants to reduce oxidative stress and liver damage. The protective effects of vitamin E provided promising results (90, 91), and subsequently the effect of vitamin E supplementation was tested in NASH adults (PIVENS trial) and NAFLD children (TONIC trial). Both studies demonstrated a favorable effect by decreasing NAS score and stimulating resolution of inflammation, but did not affect fibrosis (92, 93). Importantly, these studies used very high doses of vitamin E (400–800 IU) while the daily-recommended intake is around 22.4 IU, thereby raising some concerns regarding increased risk for unwanted side-effects. Interestingly, a recent study found an inverse correlation between vitamin E intake and serum MDA levels among women with NAFLD and NASH (39), indirectly supporting the idea to target MDA adducts in NASH. Besides vitamin E, several other approaches such as vitamin C have been proposed to reduce oxidative stress. Although vitamin C intake has been reported to be inversely correlated with NAFLD severity (94), sufficient data to support the clinical importance is still lacking and more studies in human subjects are necessary to identify modes of action to reduce reactive oxygen species and limit lipid peroxidation.

Using a transgenic *Ldlr^−/−^* mouse expressing a functional single-chain variable fragment of E06, a natural antibody capable of neutralizing OxPLs, targeting PC-OxPLs protected against several aspects of NASH, including lipid accumulation, inflammatory responses, fibrotic scarring, hepatocyte cell death, and progression to hepatocellular carcinoma, further supporting the causal role of OxPLs in the pathogenesis of NASH (47). In addition, E06 administration has been shown to protect against liver injury in a mouse model of ischemic reperfusion *via* the blockage of TLR4-mediated neutrophil activation by extracellular vesicles carrying OxPLs (95).

In line with lower anti-OSE IgM titers in human NAFLD (44), an increase in B1-derived natural IgM with specificity for OxLDL resulted in a better outcome for liver disease in atherogenic diet-fed *Ldlr^−/−^* mice deficient for the sialic acid-binding immunoglobulin-like lectin G (Siglec-G), a negative regulator of B1 cells and OSE-specific IgM. Further, since PC epitopes as found on OxLDL are also present on the cell wall of *S. pneumoniae*, cross-reactivity exists between PC epitopes from OxLDL and this microbe (96). Similar to findings in experimental atherosclerosis (97), immunization with heat-inactivated *S. pneumonia* increased specific anti-PC IgM titers and protected *Ldlr^−/−^* mice from diet-induced steatohepatitis (98). Immunized mice fed a Western-type diet presented less foamy Kupffer cells, reduced inflammatory cell infiltrates and less cholesterol crystals in the lysosomes of Kupffer cells compared to mice without immunization. Moreover, we previously showed that *in vivo* neutralization of endogenously generated MDA epitopes by intravenous administration of a specific MDA antibody (LR04) results in reduced liver inflammation in *Ldlr^−/−^* mice (45). Taken together, these studies indicate that anti-OSE IgM expansion is protective and suggest a beneficial role for B1 cells in the course of NAFLD. Therefore, identifying additional epitopes that are recognized by protective antibodies would identify novel antigens that could be useful in the development of stable peptides that might work as vaccine against NAFLD and NASH. One such example peptide might be the P1 mimotope for MDA identified by us (99). Future studies investigating the potential use of this P1 peptide in ameliorating NASH hold great promise for future applications. More studies involving human participants are needed to translate murine findings and elucidate potential protective mechanisms to identify novel treatment targets for patients with chronic fatty liver disease.

## Discussion

Evolutionary developed specialized immune recognizers of OSEs, both cellular and humoral, enables the human body to deal with key housekeeping functions such as the removal of cell debris, dying cells and damaged molecules. Heightened lipid levels and higher oxidative stress as seen during the development and progression of NASH is accompanied with increased generation and burden of lipid peroxidation end products and OSEs. Under such circumstances of excess OSE formation and/or dysfunctional removal, the immune system gets activated, generating inappropriate amounts of chemokines and pro-inflammatory cytokines, and subsequently the development and propagation of chronic inflammatory diseases such as NASH become manifest.

Of note, multiple unresolved questions remain in our understanding of the mechanisms by which OSE-induced immunity can contribute to NASH and its resolution. For instance, as bacterial dysbiosis and the role of the gut-liver axis has been widely accepted in the field of multiple chronic lipid-mediated diseases including NASH (100), one could question the potential direct effect of OxLDL and OSEs on intestinal communities. Moreover, it is not entirely clear yet how B cell-mediated antibody responses towards lipids are mediated in the gut and how these can effect liver disease manifestation. Further, as discussed in this review, although studies indicating the beneficial effect of IgM associated immune response towards OSE, all these data come from experimental mouse models. Translational studies further supporting the importance of this field in the human clinical condition during NASH are still limited. Nevertheless, it can be envisioned that gaining insights in the role of OSEs and OSE-reactive immunity in maintaining homeostasis and in controlling inflammatory responses will ultimately identify new treatment targets that can be explored to modulate NAFLD progression and inflammatory states in general.

## Author Contributions

TH and CB wrote and edited the manuscript. All authors contributed to the article and approved the submitted version.

## Funding

TH is funded by a Veni (NWO; 91619012) and Zukunftskollegs grant (FWF; ZK81B). CB is funded by the Austrian Science Fund (FWF) Project F5402-B21.

## Conflict of Interest

The authors declare that the research was conducted in the absence of any commercial or financial relationships that could be construed as a potential conflict of interest.
